# Protective Effect of Remote Limb Ischemic Perconditioning on the Liver Grafts of Rats with a Novel Model

**DOI:** 10.1371/journal.pone.0121972

**Published:** 2015-03-18

**Authors:** Junjun Jia, Jianhui Li, Li Jiang, Jing Zhang, Shasha Chen, Li Wang, Yanfei Zhou, Haiyang Xie, Lin Zhou, Shusen Zheng

**Affiliations:** 1 Key Laboratory of Combined Multi-organ Transplantation, Ministry of Public Health; Department of Hepatobiliary and Pancreatic Surgery, First Affiliated Hospital, Zhejiang University School of Medicine, Hangzhou, China; 2 Department of Anesthesia, First Affiliated Hospital, Zhejiang University School of Medicine, Hangzhou, China; University of Pecs Medical School, HUNGARY

## Abstract

**Background:**

Remote ischemic conditioning (RIC) is a known manual conditioning to decrease ischemic reperfusion injury (IRI) but not increase ischemic time. Here we tried to establish a rat RIC model of liver transplantation (LT), optimize the applicable protocols and investigate the protective mechanism.

**Methods:**

The RIC model was developed by a standard tourniquet. Sprague-Dawley rats were assigned randomly to the sham operated control (N), standard rat liver transplantation (OLT) and RIC groups. According to the different protocols, RIC group was divided into 3 subgroups （10min×3, n = 6; 5min×3, n = 6; 1min×3, n = 6）respectively. Serum transaminases (ALT, AST), creatine kinase (CK), histopathologic changes, malondialdehyde (MDA), myeloperoxidase (MPO) and expressions of p-Akt were evaluated.

**Results:**

Compared with the OLT group, the grafts subjected to RIC 5min*3 algorithm showed significant reduction of morphological damage and improved the graft function. Also, production of reactive oxygen species (MDA) and neutrophil accumulation (MPO) were markedly depressed and p-Akt was upregulated.

**Conclusion:**

In conclusion, we successfully established a novel model of RIC in rat LT, the optimal RIC 5min*3 algorithm seemed to be more efficient to alleviate IRI of the liver graft in both functional and morphological categories, which due to its antioxidative, anti-inflammation activities and activating PI3K Akt pathway.

## Introduction

**Fig 1:**
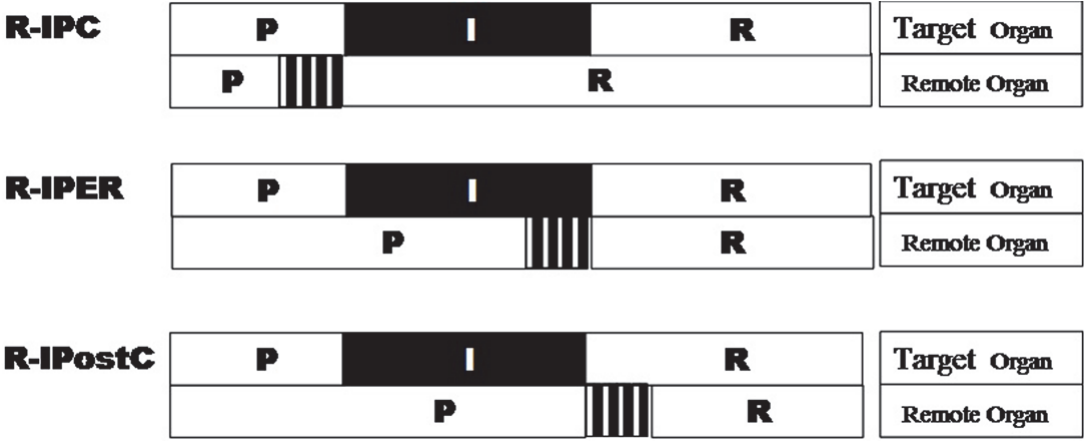
Classification of RIC according to applied time point. Note, “P” stands for “perfusion”, “I” means “ischemic”, “R” means “reperfusion”.

Nowadays, LT has developed as the universal first line treatment for the end-stage liver disease. Recent years, donation after cardiac death (DCD), domino transplantation and living donor have been increasingly applied in LT [1]. However, insufficient liver donor over the increasing demand has become one of the major existing problems. More about it, ischemic reperfusion injury (IRI) during LT is still major concerning factor in the outcome of LT [2] [3]. As we have demonstrated previously, the ischemic preconditioning (IPC) exerts powerful protective effects on attenuation of hepatic IRI by the reduction of leukocyte infiltration, hepatic enzymatic leakage and apoptotic cells formation [4, 5]. But the wide clinical application of IPC has been limited, mainly because of the unpredictability of the ischemic episode of the donors, direct mechanical trauma to major vascular structures and the potential ethical reasons [6]. Alternatively, the concept of remote ischemic conditioning (RIC) was originally developed by Przyklenk et al in 1993, showing that with the brief ischemia in one organ conferred on protection of distant important organs without direct stress to the target organ [7].The encouraging factor is, the existing research on RIC has shown the ameliorating properties on IRI of lung, intestine, kidney and heart [8–12]. Till the date, only few experimental studies have been carried out to demonstrate in the outcome of RIC on hepatectomy, focusing on brief hindlimb vascular occlusion [6, 13], infrarenal aortic clamping [14] and femoral artery occlusion [15]. Compared to IPC, RIC can be applied before ischemic event of target organ (remote ischemic preconditioning [R-IPC]), after ischemic event and before perfusion also named during ischemic event (remote ischemic perconditioning perfusion [R-IPER]) or at the onset of reperfusion (remote ischemic postconditioning [R-IPostC]) (Fig. 1) without increasing the total ischemic time. Here we mainly focus on the R-IPER which was first demonstrated effective in reducing myocardial infarction by Schimdt et al[16]. Until now, there were only 3 major studies involving the R-IPER in liver IRI which the protection function were also proved and related mechanism were investigated[17–19]. However, the effects of RIC on the liver grafts have not been reported so far. In this study we tried to establish a LT model of R-IPER, optimize the applicable protocols and investigate the potential protective mechanism in rat.

## Materials and Methods

Sprague Dawley rats (adult male, weight 250 300 g) were used in the experiment. The rats were kept in a temperature controlled environment (25 to 30°C) and supplied the standard diet water ad libitum. All procedures in this study were approved by the Ethics Committee for the Use of Experimental Animals in Zhejiang University and were carried out in accordance with the ARRIVE (Animal Research: Reporting In Vivo Experiments) guidelines (http://www.nc3rs.org/ARRIVE).

### Experimental design and groups

**Fig 2:**
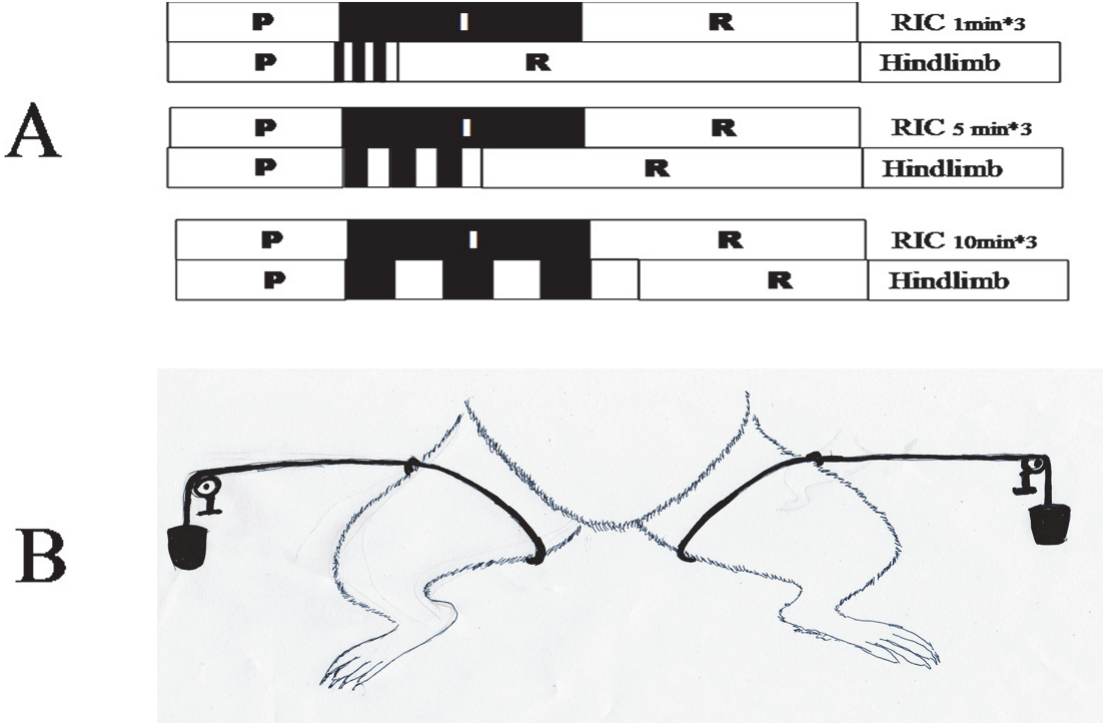
Study design and RIC model of the study. A. Details and schematic representation of RIC protocol in this study. RIC rats were divided into three subgroup (10min×3, n = 6; 5min×3, n = 6; 1min×3, n = 6). B. Schematic diagram of RIC applied in this study. Standard tourniquet was used for ligature of the hindlimb with one 1kg weight in the end.

54 Rats (24 donors) were assigned randomly to the different groups as follows, sham operated group (N, n = 6), standard rat orthotopic liver transplantation group (OLT, n = 6); RIC group (OLT with hindlimb ischemic and perfusion for different time intervals starting at the point when recipient anhepatic phase begins). According to the protocol, the RIC group was then divided into 3 subgroups （10min×3, n = 6; 5min×3, n = 6; 1min×3, n = 6）respectively (Fig. 2A).

### Rat OLT and RIC models

The OLT was performed by the method of Kamada[20] with some modification as per our research center[21]. In brief, both donor and recipient were anesthetized by intraperitoneal injection of 4% chloral hydrate anesthesia (Shanghai No. 1 Biochemical & Pharmaceutical, China). The donor was anesthetized after 12h-fasting while the recipient was done after the isolation of donor liver, then the graft was perfused through the portal vein with cold saline containing 25U/mL heparin. The graft placed into cold saline (0–4°C) for about 40 minutes before graft being transplanted into the recipient. After the completion of anastomosis of the supra-hepatic inferior vena cava followed by portal vein cuff connection, the liver was reperfused with the hepatic artery being ligated. Then the connection of infra-hepatic inferior vena cava by the cuff method, and the reconstruction of common bile duct by tying the duct over a stent was carried out.1.5 ml. Saline injected through the penile vein of the recipient after the operation. The RIC model was developed by a standard tourniquet which was performed in recipient. RIC protocols were performed by the designed cycles of reperfusion and reocclusion of the hindlimb applied immediately at the onset of recipient anhepatic phase with 1KG weight both sides (Fig. 2B). In the sham operated group, the abdomen was just opened for 75min (the mean total ischemic time during OLT in our center) and then closed under anesthesia. Upon awakening from anesthesia, rats were provided free access to sterilized water and standard rodent chow.

### Sample collection

All rats from each group were anesthetized with 4% chloral hydrate at 180 min. after OLT for the collection of samples. In detail, the rats were euthanized 3h after the portal vein of recipient was open. Blood samples were collected from the portal vein for liver function test. The liver tissue was obtained and fixed in 10% neutral formalin for the histological study purpose. The fresh liver tissue was collected and stored in −80°C for further analysis.

### Histopathologic examination

Excised liver specimens were fixed in 10% neutral buffered formalin with paraffin embedded and sectioned to 4 mm thick in accordance with our standard protocol. The sections were gradually deparaffinized and hydrated, and examined with haematoxylin and eosin staining. Morphological assessment was performed by well experienced liver pathologist in a blind fashion. A grading scale of 0–4 was used for the histopathological assessment of IRI based on the degree of sinusoidal congestion, and necrosis of parenchymal cells according to the Suzuki classification[22] and our previous studies[23].

### Liver function and CK test

Blood samples collected were centrifuged at 3,000×g for 15 minutes in room temperature to collect the serum for liver function test and creatine kinase (CK) analysis by Hitachi 7600 automatic analyzer (Hitachi, Tokyo, Japan).

### Levels of lipid peroxidation and myeloperoxidase activity

Lipid peroxidation in liver tissue was determined by measuring the formation of malondialdehyde (MDA), which is considered as an indirect measurement of oxidative damage induced by ROS[24], by the protocols of manufacturer (Nanjing Jiancheng Bioengineering Institute, Nanjing, China). All assays were performed with Bechman Coulter DU-800 Spectrophotometer (USA). As a marker of polymorphonuclear neutrophil (PMN) infiltration[25], the activity of myeloperoxidase (MPO) in hepatic tissue was measured with MPO assay kit (Nanjing Jiancheng Bioengineering Institute) in accordance with the instructions provided by manufacturer.

### Measurement of P-Akt expression in liver graft

Protein was extracted from liver by the method as described previously [26] and quantified with the Bradford assay. Lysate protein was separated by 10% SDS-PAGE gels and then transferred to nitrocellulose membranes (Bio-Rad Laboratories). After blocking with 5% fetal bovine serum in tris-buffered saline with tween (10 mmol/L Tris-HCl, 0.15 mol/L NaCl, and 0.05% Tween 20, pH 7.2), blots were incubated with the rabbit polyclonal anti- P-Akt antibody (1: 1000; Signalway antibody, USA) or mouse monoclonal anti-β-actin antibody (1: 1000; Dawenbioscience, China) for 16 h at 4°C. Blots were washed and incubated with HRP-conjugated anti-rabbit or anti-mouse secondary antibodies (1:2000; Dawenbioscience, China) for 1.5 h. Western blots were developed with the enhanced chemiluminescence system (ECL kit; Pierce Biotechnology, Rockford, IL, USA) and captured on light-sensitive imaging film.

### Statistical analysis

All experimental data are shown as mean ± SD. Statistical differences were calculated with one-way analysis of variance (ANOVA) followed by the Dunnett’s test for multiple comparisons or by Student t test for the histopathological outcomes. A p<0.05 was considered to be statistically significant.

## Results

All operations were successful. During the 3h observation, no death and other complications such as infection and embolism et al were found. All the following outcome points were at 3h after the portal vein of recipient was open.

### Optimal RIC algorithm improves the liver histopathological outcomes

**Fig 3:**
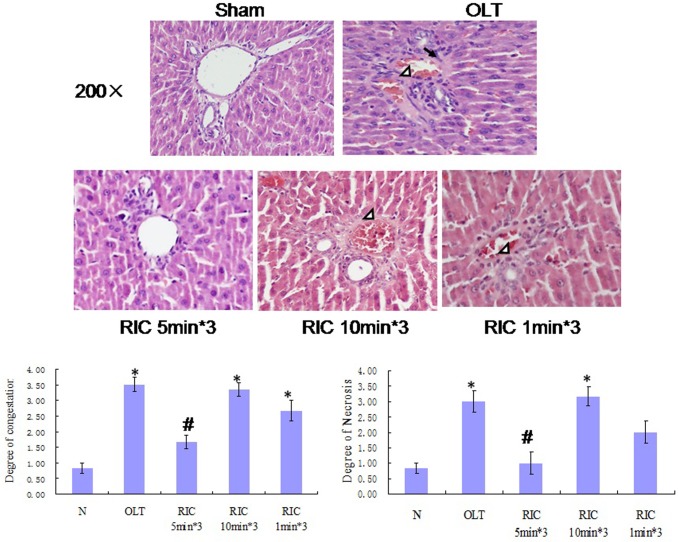
Liver histopathological presentations of all groups (Haematoxylin and Eosin stain, original magnification ×200). Hepatocyte necrosis (arrow) and congestion of the sinusoids and central vein (white arrow head). (Values are mean (s.e.m.) * p<0.05 compared with sham, # p<0.05 compared with OLT group).

Comparing with N group, sinusoidal congestion of all other groups is elevated. Comparing with OLT, RIC 5min*3 diminished the sinusoidal congestion (P = 0.001) while in RIC 10min*3 and RIC 1min*3 are not. Among RIC groups, RIC 5min*3 has lower sinusoidal congestion than RIC 10min*3 (P = 0.002) while almost the same as RIC 1min. RIC 10min*3 shows severe congestion compared with RIC 1min*3. (Fig. 3)

Comparing with N group, multifocal areas of coagulated hepatic cellular necrosis level of all other groups are elevated. Comparing with OLT group, multifocal areas of coagulated hepatic cellular necrosis in RIC 5min*3 diminished (P = 0.026) while in RIC 10min*3 and RIC 1min*3 are not. Among RIC groups, RIC 5min*3 has lower hepatic cellular necrosis than RIC 10min*3 (P = 0,01) while almost the same as RIC 1min. RIC 10min*3 has more multifocal hepatic cellular necrosis compared with RIC 1min*3. (Fig. 3)

### Optimal RIC algorithm improves the liver graft function

**Fig 4:**
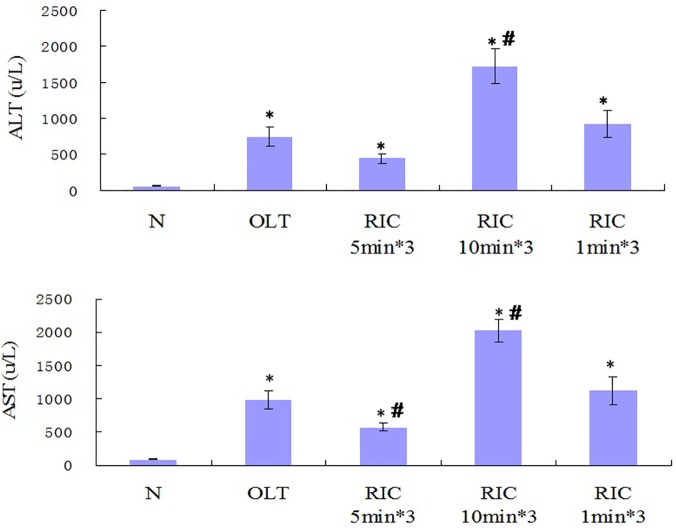
ALT and AST levels of all groups. (n = 6 in each group. Values are mean (s.e.m.) * p<0.05 *versus* with sham, # p<0.05 versus with OLT group).

Comparing with N group, Serum ALT levels of all other groups are elevated. Comparing with OLT group, elevation of ALT level is seen in RIC 10min*3 (P <0.001) but not in RIC 5min*3 and RIC 1min*3. Among RIC groups, RIC 5min*3 has the lowest ALT level (VS RIC 10min*3, P <0.001; VS RIC 1min*3, P = 0.034). RIC 10min*3 shows higher ALT compared to RIC 1min*3. (Fig. 4)

Comparing with N group, Serum AST levels of all other groups are elevated. Compared with OLT group, increase in the AST level is found in RIC 10min*3 further (P <0.001) while obvious decrease in AST level in RIC 5min*3 (P = 0.047) but remains almost same in RIC 1min*3. Among RIC groups, RIC 5min*3 reported the lowest AST level (VS RIC 10min*3, P <0.001; VS RIC 1min*3, P = 0.011). RIC 10min*3 shows higher AST compared to RIC 1min*3. (Fig. 4)

### Optimal RIC algorithm decreases MDA and MPO

**Fig 5:**
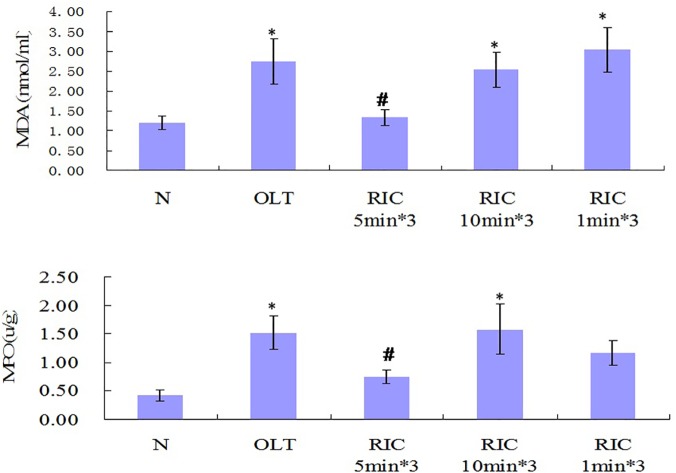
MDA and MPO levels of all groups. (n = 6 in each group. Values are mean (s.e.m.) * p<0.05 *versus* with sham, # p<0.05 versus with OLT group).

Comparing with N group, MDA levels of all other groups are elevated. Compared with OLT group, RIC 5min*3 diminished the MDA level (P = 0.006) while not in RIC 10min*3 and RIC 1min*3. Among RIC groups, RIC 5min*3 shows the lowest MDA level (VS RIC 10min*3, P = 0.014; VS RIC 1min*3, P = 0.002). RIC 10min*3 had lower MDA compared to RIC 1min*3. (Fig. 5)

Comparing with N group, MPO levels of all other groups are elevated. Comparing with OLT group, markedly decrease in MPO level can be noticed in RIC 5min*3 (P = 0.002) but not in RIC 10min*3 and RIC 1min*3. Among RIC groups, RIC 5min*3 shows the lowest MPO level (VS RIC 10min*3, P = 0.002; VS RIC 1min*3, P = 0.022). RIC 10min*3 shows higher MPO compared to RIC 1min*3. (Fig. 5)

### Optimal RIC algorithm does not increase CK

**Fig 6:**
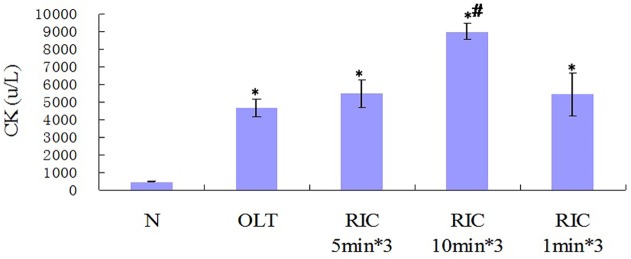
CK levels of all groups. (n = 6 in each group. Values are mean (s.e.m.) * p<0.05 *versus* with sham, # p<0.05 versus with OLT group).

Comparing with N group, Serum CK levels of all other groups are elevated. Compared to OLT group, CK level is increased in RIC 10min*3 (P <0.0001) while not in RIC 5min*3 and RIC 1min*3. Among RIC groups, RIC 5min*3 shows lower CK level than RIC 10min*3 (P = 0.002) while the same as RIC 1min. RIC 10min*3 shows higher CK compared to RIC 1min*3. (Fig. 6)

### Optimal RIC algorithm up-regulates the PI3K-Akt protein expressions

**Fig 7:**
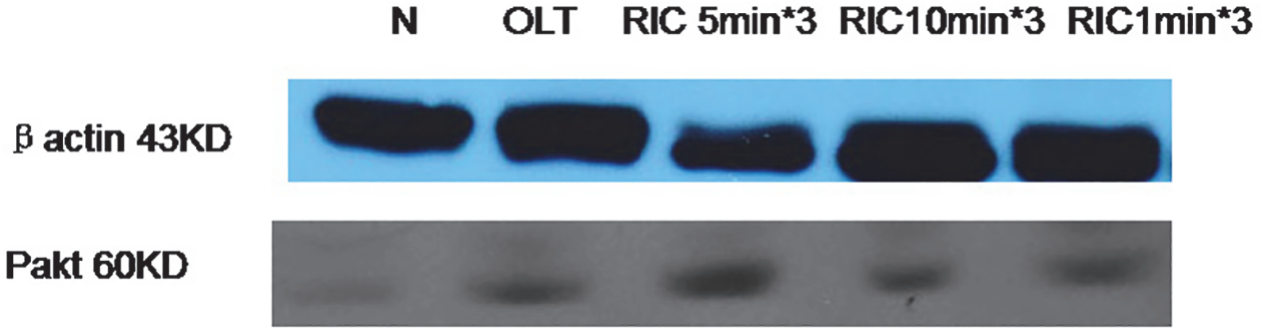
Expression of Pakt of all groups.

The protein expression levels of PI3K Akt were measured by western blot analysis. The results revealed that the expression levels of PI3K Akt in the liver tissues were significantly increased in the RIC 5min*3 compared to OLT. (Fig. 7)

## Discussion

According to our knowledge, it was first reported model of RIC in LT. No study in R-IPER comparing different times and protocols of limb occlusion was searched in Medline. This model was easily developed by a standard tourniquet applied immediately at the onset of recipient anhepatic phase with 1KG weight both sides (Fig. 2B), which did not increase CK level, in another word no further local mechanism trauma is induced. Besides, R-IPER especially 5min*3 markedly attenuated the IRI, improved liver function and reduced inflammation reaction, verifying the better outcomes in the liver graft of rats. This showed this model is easily developed, effective and particle.

Compared to the local ischemic conditionings (pre and post ischemic conditioning), RIC overcomes the main limitation of increasing total ischemic time which may bring sequence problems. Similarly to local ischemic conditionings, also there are three main components of the RIC algorithm: the duration of the initial reperfusion phase, the duration of each subsequent ischemia and reperfusion cycle and the number of cycles applied[27]. Some studies have proved that RIC could reduce IRI in small animal liver ischemia models [14, 13], however, according to our knowledge there is limited research so far to prove the effects of R-IPER on the liver graft[28]. 10min protocol[13] and 5min protocol [28] were showed protective role against hepatic IRI and enhanced the early antioxidative activity. In this study, we compared different R-IPER algorithm by selecting alternating cycles of brief reperfusion interrupted by brief ischemia starting at the beginning of anhepatic phase. It showed that only R-IPER 5min*3 induced the protective effects for the liver grafts. In all the groups, the R-IPER 10min*3 protocol showed the less protective effect, even worse than OLT for the indexes of ALT, AST and CK, Which is not the same as previous result[13].The possible reasons are related to the different animal model and the different weight used for the hind limb. On the other side, R-IPER 1min*3 was found almost the same with OLT, this may due to the stimulus of this protocol was not strong enough to induce the protective effect. So based on the observation, we can conclude that selection of the duration of ischemia and optimizing the protective protocol algorithm maybe associated to the degree of protection and can impact on the outcome of remote ischemic conditioning. The R-IPER 5min*3 protocol leads to more perceptible liver protection. This keeps in line with previous reports which 5min*4 were applied [17–19]. This finding provides the further evidence for the application of optimized RIC protocol in liver graft model.

Results from previous studies demonstrated the IRI suppression effects of RIC are through the multiple mechanisms [15, 29], though they are still not clarified yet. One of the major mechanism now known is to intervene the cascade of oxidative-stress-related events [30]. In present experiments, the protective effects have also been introduced in the OLT model of rat, which provides the further evidence that RIC protocol was feasible and practicable to apply in the OLT of rat model.

Our results showed the R-IPER 5min*3 exerted the protective effect on the morphological levels by attenuating the congestion and the hepatocyte necrosis (Fig. 3).On the functional level, it is demonstrated that all the groups except N group showed a significant increase of the ALT and AST. Conversely, the R-IPER 5min*3 dramatically improved in the protection of the grafts by inhibiting the increase in ALT and AST levels, which are the crucial biochemical parameter reflecting the function of liver. These results indicated there may be a component of morphological and biochemical adaptation of the graft to ischemia after RIC. In present study, R-IPER especially 5min*3 resulted in a marked reduction of lipid peroxidation product, MDA formation, and suppression of the infiltration of neutrophils causing to decrease the MPO level, which suggested that these protective effects may be associated with its enhanced capacities of antioxidant and anti-inflammatory activities with subsequently improvement in sinusoidal endothelial function following hepatic IRI of OLT.

Evidence showed PI3-kinase and its downstream effector protein kinase B (Akt) as well as eNOS are known to play an important role against the reperfusion injury by regulating cellular activation, inflammatory responses and apoptosis[31]. As the redox-sensitive kinase, PI3-kinase/Akt may be activated by site-specific phosphorylation, when intracellular ROS levels changed, further result in eNOS activation and increased reperfusion NO generation, and subsequently exert the cytoprotective effect[32]. In present experiment, compared to control group, applying the R-IPER 5min*3 protocol significantly enhanced Akt phosphorylation at 3 hours after reperfusion in the liver graft. This study has demonstrated that RIC protected the liver graft of rat against IRI by increasing the activities of PI3K Akt, which is crucial for the protective effects against IRI in the graft liver of rat. However the further study for the detail role of P-Akt for this protective effect is still needed.

In conclusion, we successfully established a novel model of RIC in rat LT, the optimal R-IPER 5min*3 algorithm seemed to be more efficient to alleviate the detrimental effects of IRI of the liver graft in both functional and morphological categories, which depended on its antioxidative, anti-inflammation activities and activating PI3K Akt pathway. The strategy of R-IPER 5min*3 is relatively simple to perform, particularly during LT, and may be considered as a potentially applicable in the clinical field.

## Supporting Information

S1 Certificate Certification of language correction. (PDF)

S1 Dataset Original data for this study. (XLS)
